# A mixed methods study to assess the effectiveness of food-based interventions to prevent stunting among children under-five years in Districts Thatta and Sujawal, Sindh Province, Pakistan: study protocol

**DOI:** 10.1186/s12889-016-3976-y

**Published:** 2017-01-05

**Authors:** Sumra Kureishy, Gul Nawaz Khan, Shabina Arrif, Khizar Ashraf, Angela Cespedes, Muhammad Atif Habib, Imtiaz Hussain, Asmat Ullah, Ali Turab, Imran Ahmed, Shehla Zaidi, Sajid Bashir Soofi

**Affiliations:** 1 Department of Paediatrics and Child Health, Aga Khan University, Karachi, Pakistan; 2 World Food Programme, Islamabad, Pakistan; 3 Department of Community Health Sciences, Aga Khan University, Karachi, Pakistan

**Keywords:** Food-based, Interventions, Effectiveness, Stunting, Prevention, Pakistan

## Abstract

**Background:**

Maternal and child malnutrition is widely prevalent in low and middle income countries. In Pakistan, widespread food insecurity and malnutrition are the main contributors to poor health, low survival rates and the loss of human capital development. The nutritional status trends among children exhibit a continuous deteriorating with rates of malnutrition exceeding the WHO critical threshold. With the high prevalence of maternal and child malnutrition, it is important to identify effective preventative approaches, especially for reducing stunting in children under-five years of age. The primary aim of this study is to assess the effectiveness of food-based interventions to prevent stunting in children under-five years.

**Methods:**

A mixed methods study design will be conducted to evaluate the effectiveness of food-based interventions to prevent stunting among children under-five years in districts Thatta and Sujawal, Sindh Province, Pakistan. The study will include cross sectional surveys, a community-based cluster randomized controlled trial and a process evaluation. The study participants will be pregnant women, lactating mothers and children under-five years. The cross-sectional surveys will be conducted with 7360 study participants at baseline and endline. For the randomized control trial, 5000 participants will be recruited and followed monthly for compliance of food-based supplements, dietary diversity, pregnancy outcomes, and maternal and child morbidity and mortality. Anthropometric measurements and hemoglobin levels will be measured at baseline, quarterly and at endline. The interventions will consist of locally produced lipid-based nutrient supplement (Wawamum) for children 6–23 months, micronutrient powders for children 24–59 months, and wheat soya blends for pregnant and lactating mothers. Government lady health workers will deliver interventions to participants. The effectiveness of the project will be measured in terms of the impact of the proposed interventions on stunting, nutritional status, micronutrient deficiencies, and other key indicators of the participants. The process evaluation will assess the acceptability, feasibility and potential barriers of project implementation through focus group discussions, key informant interviews and household surveys. Data analysis will be conducted using STATA version 12.

**Discussion:**

There is considerable evidence on the effectiveness of food-based interventions in managing stunting in developing countries. However, these studies do not account for the local environmental factors and widespread nutrient deficiencies in Pakistan. These studies are often conducted in controlled environments, where the results cannot be generalized to programs operating under field conditions. The findings of this study will provide sufficient evidence to develop policies and programs aimed to prevent stunting in children 6–59 months and to improve maternal and child health and growth outcomes in poor resource settings.

**Trial registration:**

NCT02422953. Registered on April 15, 2015.

## Background

Maternal and child malnutrition, also known as undernutrition, consists of stunting, wasting, underweight and micronutrient deficiencies [1, 2]. On a global scale, malnutrition is widely prevalent in low-income and middle-income countries (LMICs) [2, 3]. It is estimated to contribute to high levels of morbidity and mortality in children (6–59 months), pregnant and lactating mothers (PLWs) [3]. Malnutrition has an adverse effect on the survival, healthy development, rate of acute and chronic diseases and economic productivity of individuals, communities and societies. Maternal and child malnutrition contribute to poor growth and development of children leading to long-term effects into adulthood [3].

Maternal malnutrition, assessed through low body-mass index (<18.5 kg/m^2^), has decreased slightly over the past two decades, but still remains higher than 10% in Asia and Africa [3]. It is associated with fetal growth restriction (IUGR), which augments the risk of neonatal mortality and stunting by 24 months of age. Annually, it is estimated that 45% (3.1 million) of all child deaths are related to malnutrition, including IUGR, suboptimal breast feeding, stunting, wasting, and vitamin A and zinc deficiencies [3–5]. In 2013, 161 million children under 5 years of age globally were stunted as a consequence of chronic malnutrition and an additional 51 million were wasted due to acute malnutrition [4]. A total of 157 000 and 116 000 child deaths, in 2011, were attributed to deficiencies of vitamin A and zinc, respectively. Iron and iodine deficiencies were associated with poor child development and adverse effects on pregnancy, especially maternal mortality [3, 6]. Therefore, improving nutrition is essential to improving health, with a focus on the first 1000 days of life, pregnant and lactating women [4].

In Pakistan, widespread food insecurity and malnutrition are the main contributors to poor health, low survival rates and the loss of human capital development [7]. Despite sufficient production of staple foods nationally and an increased caloric intake per capita, no major change has been seen in the prevalence of malnutrition over the past 20 years [7, 8]. Instead, trends in nutritional status among children exhibit a continuous deteriorating with rates of malnutrition exceeding the WHO critical threshold (15%). In 2011, it was estimated that 45% of children under five were stunted, 11% were wasted and 30% were underweight [9]. As for micronutrient deficiencies, children under five were reported to have deficiencies of vitamin A (56%), vitamin D (41.1%), zinc (36.5%), iodine, and suffer from iron deficiency anemia (33.4%) [7]. Additionally, a total of 18% of Pakistani women (15–49 years old) were thin (low BMI) and undernourished; while 5% of women were short statured (<145 cm). The presence of micronutrient deficiencies is a common health problem among PLWs. Among these women, the common micronutrient deficiencies are vitamin A (48.8%), zinc (48.3%), calcium (58.3%), vitamin D (85.1%) and iron deficiency anemia (25.9%) [7].

The common determinants of malnutrition are poverty, lack of purchasing power, lack of access to nutritious food, household food insecurity, suboptimal breastfeeding, poor complementary feeding practices, childhood infections and low maternal education [9–11]. In recent years, natural disasters have led to a further increase in malnutrition due to food insecurity. It is estimated that 58% of households are food insecure [9]. Household food insecurity has led children under two years of age to consume less than half of their daily energy requirements and a lower than recommended level of micronutrients. In 2013, less than 4% of children were identified as receiving an adequate, acceptable and diverse diet [9]. Suboptimal breastfeeding is associated with increased risk of mortality in the first two years of life [3]. Poor feeding practices in infant and young children have a direct impact on child survival. To lower the risk of these adverse effects, mothers are recommended to exclusively breastfeed newborns for the first 6 months with early initiation within the first hour of life [12, 13]. After 6 months, infants should receive complementary foods with continued breastfeeding up to 2 years of age [12, 13]. According to the National Nutrition Survey, 65% of mothers exclusively breastfed for 6 months; with 40.5% practicing early initiation within the first hour of birth [7]. A total of 78% of mothers continued to breastfeed up to 12–15 months, and 52% of mothers started complementary foods as of 6–8 months. The rates of stunting, wasting and undernutrition in children (6–59 months) are higher among poorer households and mothers with lower education and low BMI [7].

There are many different methods to address malnutrition through food-based interventions in developing countries. Some commonly used food-based interventions are lipid-based nutrient supplements (LNS), fortified blended foods (FBFs) and micronutrient powders (MNPs). When provided to malnourished children, LNS were seen to modestly improve weight gain, weight-for-height, and mid-upper arm circumference (MUAC) [14]. In children under five years of age, LNS increased the rate of recovery by 10% and decreased the rate of non-recovery by 47% when compared to FBFs. In addition, FBFs – corn-soy blend (CSB), wheat soya blend (WSB) and locally produced blended foods – resulted in similar beneficial outcomes as seen with LNS. A systematic review of MNPs revealed a reduction in retinol deficiency by 21%, anemia by 34% and iron deficiency anemia by 57% in women and children [15].

There is considerable evidence on the effectiveness of food-based interventions in managing stunting in developing countries [10]. However, these studies do not account for the local environmental factors and widespread nutrient deficiencies in Pakistan. These studies are often conducted in controlled environments, where the results cannot be generalized to programs operating under field conditions. Hence, there is a need for operational research of food-based interventions to determine the impact, advantages and optimal length of supplementation for children (6–59 months), and PLWs.

## Methods

### Study design

A mixed methods study design will be used to evaluate the effectiveness of food-based interventions to prevent stunting among children under-five years in districts Thatta and Sujawal, Sindh Province, Pakistan. The study will consist of cross sectional surveys, a community-based cluster randomized controlled trial (cRCT) and a process evaluation.

### Objectives

Our primary objective is to test the hypothesis that food-based interventions will reduce stunting among children (6–59 months) by 5% annually. Our secondary objectives are to assess the effect of food-based interventions on micronutrient deficiencies in children, wasting and anemia in PLWs and reduction in low birth weight (LBW).

### Study setting

This study will be conducted in the Thatta and Sujawal Districts of Sindh Province, Pakistan. Within these districts, 29 Union Councils (UCs) with the best performance and highly covered Lady Health Workers (LHWs) catchment areas will be selected as the intervention clusters. Of the 29 Union Councils, the areas without LHWs coverage will be selected as the control clusters.

### Study population

The study participants will be PLWs and children 6–59 months of age. The LHW family register will be used to identify households and recruit participants for the intervention clusters. For study control clusters, a social mapping approach will be used, where data collection teams will meet with health facility officials and community members (elders, mothers, dias, midwives, and social workers) to identify households with PLWs and children.

### Sample size & sampling strategy

Cross-sectional Surveys: The sample size was calculated for comparing two cross-sectional surveys, while assuming the baseline prevalence of stunting to be 49% with a 10% reduction in the prevalence of stunting at the end [16]. For estimating impact of the interventions (α = 0.05, power = 0.80), each group should have 3200 participants. To account for dropouts and data errors, the sample size was increased by 15% giving a new total of 7360 study participants (3680 participants per cluster).

A two-staged cluster sampling technique will be used to select study participants. Within the 29 UCs, LHW catchment areas will be used as clusters for the intervention group, while the remaining uncovered areas will be used for the control group. As previously mentioned, all households with PLWs and children 6–59 months of age will be eligible for the baseline and endline surveys. Eligible participants will be selected randomly from a complete household listing created using the LHW family register and social mapping approach.

Cluster Randomized Controlled Trail (RCT): For the cluster RCT, the main outcome is the prevention of stunting among children 6–59 months in a community setting. UCs will be used as clusters. The sample size for randomized clusters was calculated using a two-sample comparison of means to detect a 0.10 difference between groups, with a standard deviation of 1.00 and an intracluster correlation of 0.0001 [17]. For a two-tailed test (α = 0.05, power = 0.85), each group will require 5 clusters with 500 participants per cluster. Of the 29 UCs, a total of 10 UCs will be selected using simple random sampling. There will be 5 UCs in the intervention group and 5 UCs in the control group, giving a total sample size of 5000 participants. Due to the nature of the intervention, blinding will not be possible.

Process Evaluation: To collect quantitative data for the process evaluation, household surveys will be conducted at baseline, six-monthly and endline. Furthermore, qualitative data will be collected through 18 in-depth interviews (IDIs) with key informants and 18 focus group discussions (FGDs) with PLWs at baseline and endline. At six-monthly intervals, 12 FGDs will be conducted with PLWs and 30–40 interviews with LHWs. Key informants will be selected from each district using a snowballing approach. Six intervention clusters will be randomly selected for the FGDs. Subsequently, from each cluster one village will be selected to recruit participants for the FGDs.

### Interventions

A comprehensive package of community-based interventions will be implemented to address stunting among children (6–59 months). The interventions will focus on food-based supplementation and non-food based interventions delivered through LHWs. A blanket approach will be used for the distribution of food-based supplementation, consisting of locally produced LNS for children 6–23 months, MNPs for children 24–59 months and WSB for PLWs. The control group will receive routine public and private health services available within the area.

Lipid-based Nutrient Supplement (LNS): LNS are called lipid-based because a large percentage of their calories come from fats (peanuts, milk, and vegetable oils) [18]. They also contain a full complement of vitamins and micronutrients. A locally produced LNS (Wawamum) made from chickpeas will be distributed to children aged 6–23 months. A daily ration of 50 g of Wawamum will be provided to cover the RDA of most micronutrients and about 1/4 of daily energy requirements.

Micronutrient Powders (MNPs): MNPs are single-dose packets with vitamins and minerals in powder form that can be added to any semi-solid food at home or any other place [19]. It is used to help increase micronutrient intake without alternating the dietary habits of children. On alternate days, children aged 24–59 months will receive a MNPs sachet to obtain the RDA of 15 micronutrients.

Wheat Soya Blend (WSB): WSB are partially cooked cereals, pulses, soya and beans, which are fortified with vitamins and minerals [20]. A monthly ration of 5 kg of WSB will be provided to pregnant women during pregnancy and for first 6 months to lactating mothers.

Behaviour Change Communication: Behavior change and preventative health education messages will be provided on product use and benefits, infant and young child feeding practices (IYCF) and maternal nutrition by LHWs using group sessions and home visits. The nutrition education will be based on the material developed by WFP and the Health Sector.

### Data collection & measurements

Cross Sectional Surveys: A structured household survey will be used to collect data on socioeconomic status and nutrition-related indicators. The surveys will be administered at baseline and endline. The indicators assessed will be family size, household expenditures, IYCF practices, child health status, child morbidity, food consumption and diet diversity. Informed consent will be obtained from all participants prior to the collection of data.

Anthropometric Measurements: All participants will undergo anthropometric measurements such as height, weight and mid upper arm circumference (MUAC). The measurements will be taken at baseline, quarterly follow-ups and endline. For children, the measurements of length/height-for-age, weight-for-age and weight-for-length/height will be extrapolated using the collected data. Age will be determined using birth certificates, immunization cards, or other documentation. In the absence of documentation, a local calendar will be used to determine age to the nearest month.

Hemoglobin Testing: A spot hemoglobin test will be conducted to assess the prevalence of anemia among participants using a HemoCue machine. The test will be conducted at baseline and endline.

Blood Sample Collection: Blood samples will be collected twice from a subset of 200 children (100 intervention group, 100 control group) within the cluster RCT cohort. About 3 ml of blood will be obtained from the children at 6–8 months of age and subsequently at 24 months of age. The blood samples will help to assess the impact of the intervention on micronutrient deficiencies.

Monthly Follow-ups in RCT: Monthly follow-ups in RCT will be conducted to assess compliance to the intervention, dietary diversity, pregnancy outcomes, and maternal and child morbidity and mortality. Food compliance will be measured using parental recall and observation of used and available sachets. The follow-ups will help monitor the impact of the intervention on the linear growth of children.

Process Evaluation: A process evaluation will be conducted to assess the implementation and expected outputs of the interventions. As a continuous strategy, the evaluation will require data to be collected at baseline, six-monthly basis and endline. For qualitative data collection, trained moderators will conduct the interviews in Sindhi, the local language spoken in Thatta and Sujawal. During the interviews, a note taker, moderator, and observer will be present as per study protocol.

Data Quality Control & Quality Assurance: A separate team of trained female data collectors will revisit the 5% of households for the purpose of data quality control and quality assurance. The data collection process will be supervised and monitored by field supervisor and project manager. In addition, the team leaders will review the questionnaires for completeness, while the field supervisor will review for errors and inconsistencies. In the presence of errors or inconsistencies, the questionnaires will be returned to the data collectors for correction. Once reviewed, the questionnaires will be transferred to the Data Management Unit (DMU) for data entry. Data will be double entered by trained data input operators in the customizable Visual FoxPro database. ENA-SMART software will be used to track and plausibility checks for anthropometric measurements.

### Data analysis

Analysis will be conducted using univariate and multivariate methods. Descriptive statistics will be obtained to generate frequency tables on demographic data. Additionally, Pearson Chi Square Test will be conducted to establish an association between categorical variables. Bivariate analysis will be conducted to establish an association between growth factors (stunting, wasting, and underweight) and other variables. All calculations will be performed with SPSS statistical software (version 18) [21].

### Ethical consideration

Informed consent will be obtained from all participants prior to the recruitment in the study, data collection and anthropometric measurements. The consent forms will outline the objectives, measurements and process of confidentiality of the study. The Ethics Review Committee (ERC) of Aga Khan University has granted approval for the proposed trial. Furthermore, the National Bioethics Committee (NBC) of Pakistan has approved the study for human subject research.

## Discussion

The nutritional status of women during preconception, pregnancy and postpartum is critical for healthy maternal, pregnancy and birth outcomes [22]. In developing countries, women experience multiple biological and social stressors which increase their risk of malnutrition. These stressors are food insecurity, inadequate diets, poor health care, recurrent infections, heavy work burdens, gender inequalities, repeated pregnancies and short intervals between pregnancies. These stressors lead women to experience low BMI, anemia, and micronutrient deficiencies (vitamin A, zinc, iodine, folate, calcium and vitamin-D) [3, 22]. Maternal malnutrition is determined through low BMI, stunting, IUGR, and small-for-gestational-age (SGA). A low BMI is indicative of chronic energy deficiency. In 2011, the prevalence of low BMI among mothers was higher than 10% in Asia and Africa [3]. Low maternal BMI is associated with increased rates of infections, obstructed labor, LBW babies and SGA babies. LBW is a major contributor to neonatal and infant mortality; while fetal growth restriction (IUGR) contributes to stunting and wasting in children. Annually, it is estimated that IUGR occurs in 23.8% (about 30 million) of newborns [22]. The process of stunting typically begins in the fetal period, resulting in approximately one fifth of childhood stunting [23]. Hence, the process highlights the importance of improving maternal nutritional status through food-based interventions.

In developing countries, child malnutrition is a major challenge leading to disastrous health outcomes [22]. Malnutrition in children is assessed through IUGR, wasting and stunting. In children under 5, about 10% to 30% of children are affected by acute and chronic malnutrition. Research has shown that child malnutrition is attributed to a lack of appropriate childcare practices, poor diet quality and accessibility, lack of diet diversity, poverty, and recurring infections during the first 2 years of age [22, 24]. The consequences of child malnutrition are stunting, wasting, undernutrition, short adult stature, intellectual disability, reduced educational achievements, and lower earnings [22, 23]. Micronutrient deficiencies are another attributor towards child malnutrition; specifically deficiencies of vitamin A, vitamin D, calcium, iodine, zinc, folate and iron deficiency anemia [3, 23]. These deficiencies have serious effects on developing fetuses and children. Iodine deficiency may cause poor fetal brain development and stillbirths. Folate deficiency may increase the risk of neural tube defects, other birth defects and premature labour. Vitamin A deficiency and iron deficiency may increase the risk of infant morbidity and mortality, poor vision, and impaired cognitive development [23]. Food-based interventions implemented during early life to overcome child malnutrition and micronutrient deficiencies have shown a positive effect on child health and growth, human capital, and economic productivity. It is recommended that food-based interventions target the first 1000 days of life; preconception period, pregnancy, lactation and the first two years of the child’s life [23].

There is considerable evidence on the effectiveness of food-based interventions in managing stunting in developing countries. However, these studies do not account for the local environmental factors and widespread nutrient deficiencies in Pakistan. These studies are often conducted in controlled environments, where the results cannot be generalized to programs operating under field conditions. The strength of our study is that, to our knowledge, this is the first operational research study with a nested RCT that is examining the impact, advantages and optimal length of supplementation for children (6–59 months), and PLWs. The findings of this study will provide sufficient evidence to develop policies and programs aimed to prevent stunting in children (6–59 months) and to improve maternal and child health and growth outcomes in poor resource settings.

## Abbreviations

BMI: Body mass index
ERC: Ethical review committee
FGD: Focus group discussion
HAZ: Height-for-age z-scores
IDI: In-depth interview
IYCF: Infant and young child feeding practices
LBW: Low birth weight
LHW: Lady health worker
LMICs: Low-income and middle-income countries
LNS: Lipid-based nutrient supplements
MNP: Micronutrient powder
MUAC: Mid-upper arm circumference
NBC: National Bio-ethics committee
NNS: National Nutrition Survey
PDHS: Pakistan demographic health survey
PLW: Pregnant and lactating woman
PPS: Proportion to population size
SD: Standard deviation
UCs: Union councils
WAZ: Weight-for-age z-scores
WHO: Word Health Organization
WHZ: Weight-for-height z-scores
WSB: Wheat soya blends.
